# Factors associated with diabetes-related distress among Asian patients with poorly controlled type-2 diabetes mellitus: a cross-sectional study in primary care

**DOI:** 10.1186/s12875-023-02012-w

**Published:** 2023-02-27

**Authors:** Xiaoxuan Guo, Pang Nee Frida Wong, Yi Ling Eileen Koh, Ngiap Chuan Tan

**Affiliations:** 1 SingHealth Polyclinics, 167 Jalan Bukit Merah, Connection One, Tower 5, #15-10, Singapore, 150167 Singapore; 2 SingHealth-Duke NUS Family Medicine Academic Clinical Programme, 167 Jalan Bukit Merah, Connection One, Tower 5, #15-10, Singapore, 150167 Singapore

**Keywords:** Diabetes-related distress, Type 2 diabetes mellitus, Factors, Primary care

## Abstract

**Background:**

Diabetes-related distress (DRD) is a negative emotional state related to the burden of living with diabetes mellitus. It has been associated with poor self-care and glycaemic control. This cross-sectional study aimed to examine the factors associated with DRD among urban Asian patients with poorly controlled type-2 diabetes mellitus (T2DM) in primary care in Singapore. The factors included demographics, diabetes history, medical co-morbidities, mood disorders and social history.

**Methods:**

Patients with T2DM and HbA1c of 8% or more were recruited from 2 public primary care centres in Singapore. They were administered a questionnaire survey to identify DRD based on the Problem Area In Diabetes (PAID) scale. Their anxiety and depression were screened using GAD-7 and PHQ-9, and quality of life (QOL) measured using the EQ-5D-5L. Their clinical data, including HbA1c, comorbidities and medications, were extracted from the electronic medical records.

**Results:**

Among the 356 subjects, the prevalence of DRD was 17.4%. DRD was significantly associated with younger age (AOR (95% CI) = 0.93 (0.89–0.97), *p* = 0.001), ex-smoker status (AOR (95% CI) = 22.30 (2.43–204.71), *p* = 0.006) and history of kidney disease (AOR (95% CI) = 3.41 (1.39–8.35), *p* = 0.007). Those who screened positive for depression (AOR (95% CI) = 4.98 (1.19–20.86), *p* = 0.028) were almost five times more likely to have DRD. Quality of life was lower among those with DRD (EQ5D index score AOR (95% CI) = 0.11 (0.01–0.97), *p* = 0.047), who also tended to feel that diabetes pharmacotherapy interfered with their normal life (AOR (95% CI) = 2.89 (1.38–6.08), *p* = 0.005).

**Conclusion:**

About 1 in 6 patients with poorly controlled T2DM had DRD. Younger age, ex-smoker status, history of kidney disease, and those with depressive symptoms were most at risk.

**Supplementary Information:**

The online version contains supplementary material available at 10.1186/s12875-023-02012-w.

## Introduction

Type-2 diabetes mellitus (T2DM) is a challenging disease that requires commitment and adherence to a complex set of self-management tasks in order to get the best possible outcome. As a result, persons with diabetes (PWD) often feel frustrated, burnt out or overwhelmed with worries related to the current management or future implications of their disease [1]. Diabetes-related distress (DRD) refers to the negative emotional state arising from the burden of living with the disease [1]. The likelihood of DRD is higher among those with poor glycaemic control compared to those who are well-controlled [2]. Its relationship with glycaemic control is time-concordant [3]. Its presence at baseline, in one study by Aikens (2012), has been linked to future glycaemic control [4]. DRD has been associated with poorer medication adherence and lower frequencies of self-care behaviours. Both medication adherence and self-care behaviours are well-established determinants of glycaemic control, which are in turn associated with future complications and lower quality of life.

The prevalence of DRD was reported as 36%, in a meta-analysis of 55 studies, with gender and comorbid depressive symptoms as significant factors affecting prevalence [5]. However, a high level of heterogeneity was noted in these studies, and more than half of them were from the United States of America, limiting its global generalizability Distress levels and content are influenced by psychosocial, socio-economic, cultural and demographic variables, which vary across different populations. Even within the same country, prevalence of DRD differs between study sites [6] and level of care, with primary care having a lower prevalence compared to secondary care [7, 8].

Apart from gender, depressive symptoms and glycaemic control, several other factors have been identified to be associated with DRD, including younger age [6, 9–11], certain ethnicities [6, 10], living alone, lower education [9], greater number of diabetes-related complications [9, 12], frequency and severity of hypoglycaemic episodes, diabetes-related family arguments and diabetes support gap [9]. Conversely, lower levels of DRD were found with the older adults [13], married [10, 14], employed [10], higher family and social support and better patient-physician relationship [13]. Conflicting evidence was found for the association of DRD with duration of T2DM. Several studies showed its association with DRD [8, 15, 16], while one large, multinational study showed the reverse [9].

Understanding the associated factors is key to design appropriate interventions to mitigate DRD in primary care where most PWDs are managed. A systematic review by Sturt (2015) alluded that psycho-education, when conducted by general practitioners or practice nurses for six sessions or more and for three months or more, was effective in reducing DRD [17]. A chronic disease management programme in Sydney, Australia, reported the effectiveness of a diabetes health-coaching programme in reducing DRD for those with the highest level of baseline distress. It was also effective in improving the glycaemic control in those with a baseline HbA1c above the recommended level of 7% [18]. This would suggest that intervention measures would likely benefit those with a high level of distress and suboptimal glycaemic control.

Singapore has a highly urbanised multi-ethnic Asian population with the prevalence of T2DM doubling from 7.3% in 1992 to 13.7% in 2018. DRD had been identified in PWDs of a tertiary hospital but the majority of PWDs in Singapore are managed in primary care. Hence, this study aimed to assess the prevalence of DRD and its associated factors in PWDs with poor glycaemic control of HbA1c ≥ 8% in primary care. The factors included age, duration of T2DM, hypoglycaemic frequency, financial difficulties, social support and encounters with healthcare providers.

## Methods

### Subjects and study design

A cross-sectional survey using self-administered questionnaires and a retrospective health record review was conducted at two polyclinics (public primary care centres) in the northeast region of Singapore. Patients were recruited on a case-encounter basis at the patient monitoring stations within the polyclinic during their routine medical reviews.

Patients aged 40 to 79 years with T2DM on follow-up for more than 1 year, had a latest HbA1c of ≥ 8% and who could understand and comply with written and/or verbal instructions were eligible to participate in the study. While most guidelines, including the American Diabetes Association, recommend a HbA1c treatment target of < 7% for most adults, for the purpose of this study, poor glycaemic control is defined as HbA1c ≥ 8% [19]. Patients were excluded if they were on treatment for psychological disorders, mentally incapacitated or pregnant.

The questionnaire was printed in the English language, and was tested and revised in a pilot study. Interviewers were trained to use a common script to field questions, so as to ensure standardisation. For participants who spoke only Mandarin or Malay, the questionnaire was translated to the participants by interviewers who were native Mandarin and Malay speakers respectively.

Clinic staff referred patients fulfilling the eligibility criteria to a study team member, who explained the study and obtained their consent in a quiet, closed room. Following consent, the subjects filled out the questionnaire and returned it back to the study team member to check for completeness. If clarifications or translation of the questionnaire were required, the study team member would read from the standardised script based on the study protocol.

### Sample size calculation

Based on a previous study done in a local tertiary outpatient specialist clinic, the prevalence of DRD was estimated to be 32% [20]. With a 5% precision and a 95% confidence level, the sample size required for this study was calculated to be 335. This number was multiplied by 10% to allow for drop-outs and missing data, and rounded up to give a final target sample of 370.

### Study questionnaire

Data on demographics was collected directly from subjects using the questionnaire. The information included gender, ethnicity, marital status, education level, employment and living arrangements. For financial status, the Community Health Assist Scheme (CHAS), a local tiered health financing support programme, was used to identify lower- to middle-income households, while receipt of Medifund assistance (another health financing support scheme targeted at people from low socioeconomic status) was used to identify needy subjects who have difficulties with their medical bills despite government subsidies. Self-reported information on smoking history, exercise frequency, hypoglycaemic frequency and history of previous consults with a dietician and/or counsellor/psychologist was obtained from the participants.

Questions on other psychological and social factors such as diabetes-related family arguments and financial concerns were included to identify psychosocial issues. These questions were adapted from the second Diabetes Attitudes, Wishes and Needs (DAWN-2) study [9]. The latter was a global study to assess diabetes care and self-management of PWDs, family members and healthcare professionals, and to identify determinants of effective treatment and self-management [21]. Responses were recorded on a 5-point Likert scale, 5 representing “strongly agree” and 1 representing “strongly disagree”.

### Scales and tools

(i) Problem Area In Diabetes (PAID) Scale

DRD was measured using the PAID scale (Annex 1). This instrument is a 20-item questionnaire, where items are rated on a 5-point Likert scale, with 0 representing “no problem” and 4 representing “a serious problem”. The scores are summed up and multiplied by 1.25 to give a total score from 0 to 100. A PAID score of ≥40 suggests distress at a level warranting clinical attention [1]. The psychometric properties of the English version [20] as well as the Chinese translation [22] have been studied and found to be valid and reliable for use in Singapore.

In this report, the terms “DRD” and “elevated distress” will be used interchangeably to refer to a PAID score of ≥40.

(ii) Patient Health Questionnaire (PHQ-9) and Generalised Anxiety Disorder (GAD-7)

The PHQ-9 is a 9-item self-report tool used to screen for depression. Subjects are asked to rate the frequency of symptoms over the past 2 weeks on a 4-point Likert scale, with 0 representing “not at all” and 3 representing “nearly every day”. The possible score range is from 0 to 27. Scores of 5, 10, 15 and 20 represents cut-off points for mild, moderate, moderately severe and severe depression respectively [23]. The PHQ-9 has been shown to be valid and reliable (Cronbach’s α = 0.87) for use in Singapore [24].

Similarly, the GAD-7 is a 7-item self-report tool designed to screen for anxiety, where subjects rate the frequency of symptoms over the past 2 weeks on the same 4-point scale. The total score ranges from 0 to 21. Scores of 5, 10 and 15 represents cut-off points for mild, moderate and severe anxiety respectively. The scale is valid and reliable to measuring anxiety in the general population based on overseas studies [25].

A cut-off score of 10 and above was used to define cases of clinical significance for both the PHQ-9 and GAD-7.

(iii) 5-Level EuroQol 5 Dimensions Scale (EQ-5D-5L)

The EQ-5D-5L is a simple self-report survey developed by the EuroQoL Group to measure health-related quality of life [26]. It consists of two parts. The first measures health status in five domains (mobility, self-care, usual activities, pain/discomfort and anxiety/depression) across five response levels. The results may be presented as a descriptive profile or a single index value. Value sets for the latter are available for each country [27].

The second part records the respondent’s self-rated health on a visual analogue scale (EQ-VAS), ranging from 0, which represents the “worst imaginable health state”, to 100, which represents the “best imaginable health state”.

The original instrument utilises a three-level response, with the English, Chinese and Malay versions previously validated for use in Singapore [28–30]. The newer EQ-5D-5L, which saw the introduction of two new response levels to improve sensitivity and reduce ceiling effects, has been found to be more discriminative compared to the three-level version in PWDs in Singapore [31].

### Electronic medical records review

Clinical data were extracted from the electronic medical records. The data included birth year, latest body mass index (BMI) and HbA1c reading, co-morbidities and diabetes-related complications, number of doctors and nurses visits, duration of T2DM and the number of long term medications. Nursing encounters included diabetes counselling and education.

### Statistical analyses

Data analysis was performed using SPSS Version 27.0. Statistical significance was defined as *p* < 0.05. Chi-square test or Fisher’s exact test was used to analyse categorical variables. For continuous variables that are normally distributed, independent t-test and one-way ANOVA were used for two groups and three or more groups respectively. Continuous variables that are non-parametric were analysed with the Mann–Whitney U and Kruskal–Wallis tests for two groups and three or more groups respectively. Potential factors with *p*-values less than 0.2 were included in the multiple logistic regression to obtain the adjusted odds ratio (AOR).

## Results

A total of 370 patients were recruited and consented to participate in the study. 13 participants were excluded from analysis because they did not fulfil the inclusion criteria. One person dropped out mid-way through the questionnaire because of time constraint. Eventually, completed data of 356 subjects were included in the final analysis.

Patient characteristics are presented in Table 1, while disease and management data are presented in Table 2. The mean age was 58.6 years, with 50.3% female. The frequency of Chinese, Malay, Indian and Others were 62.1%, 22.5%, 13.5% and 2.0% respectively. Mean HbA1c was 9.5%, while mean BMI was 28.1 kg/m^2^. The mean duration of diabetes was 10.8 years, while the mean PAID score was 15.4. The prevalence of DRD was 17.4%.

**Table 1:** Patient demographics

	**Frequency**	**No DRD **^**b**^	**DRD **^**b**^	**Crude OR (95% CI)**	***p*-value**
Total (%)	356 (100.0)	294 (82.6)	62 (17.4)	-	-
**Age, mean (SD**^**a**^**)**	58.6 (9.5)	59.4 (9.2)	55.2 (9.8)	-	0.002
**Gender**					
Male	177 (49.7)	152 (85.9)	25 (14.1)	1	-
Female	179 (50.3)	142 (79.3)	37 (20.7)	1.58 (0.91–2.76)	0.105
**Ethnicity**					
Chinese	221 (62.1)	186 (84.2)	35 (15.8)	1	-
Malay	80 (22.5)	63 (78.8)	17 (21.3)	1.33 (0.76–2.31)	0.316
Indian	48 (13.5)	38 (79.2)	10 (20.8)		
Others	7 (2.0)	7 (100)	0 (0)		
**Marital Status**					
Married	301 (84.6)	249 (82.7)	52 (17.3)	0.94 (0.45–1.98)	0.871
Not married	55 (15.4)	45 (81.8)	10 (18.2)	1	-
**Education**					
Primary and below	115 (32.3)	98 (85.2)	17 (14.8)	0.76 (0.41–1.39)	0.367
Secondary and above	241 (67.7)	196 (81.3)	45 (18.7)	1	-
**Employment Status**					
Employed	212 (59.6)	174 (82.1)	38 (17.9)	1.09 (0.62–1.91)	0.759
Unemployed/ Retired	144 (40.4)	120 (83.3)	24 (16.7)	1	-
**Any Financial Assistance **^**c**^					
Yes	152 (42.7)	124 (81.6)	28 (18.4)	1.13 (0.65–1.96)	0.666
No	204 (57.3)	170 (83.3)	34 (16.7)	1	-

^a^ Standard deviation

^b^ PAID score < 40 indicates no distress, ≥ 40 indicates distress

^c^ Community Health Assist Scheme, Pioneer Generation Package, Medifund

**Table 2:** Disease and management characteristics

	**Frequency**	**No DRD**	**DRD**	**Crude OR (95% CI)**	***p*-value**
Total (%)	356 (100.0)	294 (82.6)	62 (17.4)	-	-
**Smoker**					
Non-smoker	280 (78.7)	228 (81.4)	52 (18.6)	5.02 (1.18–21.36)	0.029
Ex-smoker	30 (8.4)	22 (73.3)	8 (26.7)	8.00 (1.56–40.91)	0.013
Current smoker	46 (12.9)	44 (95.7)	2 (4.3)	1	-
**Exercise**					
None/Irregular	292 (82.0)	240 (82.2)	52 (17.8)	1.17 (0.56–2.45)	0.677
Regular ^a^	64 (18.0)	54 (84.4)	10 (15.6)	1	-
**Clinical parameters and medical conditions**					
**BMI, mean (kg/m**^**2**^**)**	28.1 (6.1)	28 (6.1)	29 (5.7)	-	0.223
**HbA1c, mean (%)**	9.5 (1.5)	9.4 (1.5)	9.7 (1.4)	-	0.242
**Duration of diabetes, mean (years)**	10.8 (7.2)	10.9 (7.4)	10.3 (6.1)	-	0.511
**Experience symptoms of low blood sugar in past 12 months**					
None	266 (74.9)	228 (85.7)	38 (14.3)	1	-
At least once every few months	89 (25.1)	65 (73)	24 (27)	2.18 (1.22–3.90)	0.008
**Hypertension**					
Yes	318 (89.3)	263 (82.7)	55 (17.3)	0.93 (0.39–2.21)	0.863
No	38 (10.7)	31 (81.6)	7 (18.4)	1	-
**Dyslipidemia**					
Yes	343 (96.3)	282 (82.2)	61 (17.8)	2.60 (0.33–20.34)	0.364
No	13 (3.7)	12 (92.3)	1 (7.7)	1	-
**Nephropathy and/or Chronic kidney disease**					
Yes	237 (66.6)	188 (79.3)	49 (20.7)	2.13 (1.10–4.10)	0.024
No	119 (33.4)	106 (89.1)	13 (10.9)	1	-
**Number of polyclinic doctors’ visits for DM in the past 12 months**	5.1 (1.7)	5.1 (1.7)	5.2 (1.6)	-	0.756
**Has a regular doctor for diabetes (i.e. ≥ 2 visits with the same doctor in the past 12 months**					
Yes	175 (49.2)	146 (83.4)	29 (16.6)	1	-
No	181 (50.8)	148 (81.8)	33 (18.2)	1.12 (0.65–1.94)	0.680
**Number of polyclinic nurses’ encounters related to DM within the past 12 months**	2 (1.7)	2 (1.7)	2 (1.8)	-	0.817
**Were there previous nurse encounters related to DM before the past 12 months?**					
Yes	281 (78.9)	231 (82.2)	50 (17.8)	1	-
No	75 (21.1)	63 (84.0)	12 (16.0)	0.88 (0.44–1.75)	0.716
**Total number of ALL active regular medications**	6.5 (2.4)	6.5 (2.4)	6.4 (2.2)	-	0.752
**Total number of DM medications**	2.7 (0.9)	2.7 (0.9)	2.8 (0.8)	-	0.445
**Number of comorbidities**	3.2 (1.2)	3.2 (1.2)	3.3 (0.9)	-	0.840
**Consulted dietician recently**					
Yes (within 1 year)	41 (11.5)	32 (78.0)	9 (22.0)	1.39 (0.63–3.08)	0.417
No	315 (88.5)	262 (83.2)	53 (16.8)	1	-

^a^ ≥ 5x/week AND ≥ 30 min/day

Subjects with DRD were younger than those without DRD (55.2 years old vs 59.4 years old, *p* = 0.02) (Table 1). No difference in duration of illness was noted between the two groups (Table 2).

Those who experienced symptoms of hypoglycaemia were more likely to have DRD compared to those without such symptoms (OR (95% CI) = 2.18 (1.22–3.90), *p* = 0.008). Non-smokers and ex-smokers were more likely to have DRD compared to current smokers (OR (95% CI) = 5.02 (1.18–21.36), *p* = 0.029 and OR (95% CI) = 8.00 (1.56–40.91), *p* = 0.013). Subjects with kidney disease were also more likely to have DRD (OR (95% CI) = 2.13 (1.10–4.10), *p* = 0.024). No difference was associated with other co-morbidities such as hypertension, dyslipidaemia, ischemic heart disease, cerebrovascular disease, diabetic eye disease, peripheral vascular disease, neuropathy, diabetic foot disease and anaemia. Financial assistance (Table 1), or previous diabetes-related nurse encounters or previous dietician encounters were not associated with DRD (Table 2).

Those screened positive for anxiety and depression using GAD-7 and PHQ-9 with scores of 10 or higher were more likely to have DRD (OR (95% CI) = 16.77 (6.62–42.46) and OR (95% CI) = 23.14 (7.36–72.72), both *p* < 0.001) (Table 3).

**Table 3:** Psychological screeners and quality of life measure

	**Frequency**	**No DRD**	**DRD**	**Crude OR (95% CI)**	***p*-value**
Total (%)	356 (100.0)	294 (82.6)	62 (17.4)	-	-
**PAID score, mean (SD)**	15.4 (15.4)	9.6 (8.5)	43 (9.3)	-	< 0.001
**GAD-7 score, mean (SD)**	2.5 (4)	1.5 (2.6)	7.3 (5.8)	-	< 0.001
**GAD-7 score**^**a**^					
< 10	331 (93)	287 (86.7)	44 (13.3)	1	-
≥ 10	25 (7)	7 (28)	18 (72)	16.77 (6.62–42.46)	< 0.001
**PHQ-9 score, mean (SD)**	2.7 (3.6)	2 (2.4)	6.3 (5.7)	-	< 0.001
**PHQ-9 score**^**b**^					
< 10	337 (94.7)	290 (86.1)	47 (13.9)	1	-
≥ 10	19 (5.3)	4 (21.1)	15 (78.9)	23.14 (7.36–72.72)	< 0.001
**EQ VAS**	73.9 (17.3)	75.4 (16.9)	66.6 (17.3)	-	< 0.001
**EQ5D Index**	0.9 (0.2)	0.9 (0.2)	0.8 (0.2)	-	< 0.001

^a^ GAD-7 score ≥ 10 defines anxiety of clinical significance

^b^ PHQ-9 score ≥ 10 defines depression of clinical significance

Subjects with DRD had a lower mean EQ-VAS score compared to those without DRD (66.6 vs 75.4, *p* < 0.001), and a lower EQ-5D index score compared to those without DRD (0.8 vs 0,9, *p* < 0.001) (Table 3).

Subjects who felt anxious about their weight, felt discriminated against, found difficulties paying for medications, worried about their financial future and felt that their medications interfered with their normal life were more likely to have DRD (Table 4).

**Table 4:** Other Psychological and social factors

	**Freq**	**No DRD**	**DRD**	**Crude OR 95% CI)**	***p*-value**
Total (%)	356 (100.0)	294 (82.6)	62 (17.4)	-	-
**I feel anxious about my weight**					
Not sure	17 (4.8)	12 (70.6)	5 (29.4)	3.27 (1.75–6.09)	< 0.001
Strongly agree/ Agree	165 (46.3)	124 (75.2)	41 (24.8)	4.11 (1.29–13.17)	0.017
Strongly disagree/ Disagree	174 (48.9)	158 (90.8)	16 (9.2)	1	-
**I feel discriminated against because I have diabetes**					
Not sure	12 (3.4)	7 (58.3)	5 (41.7)	5.00 (2.25–11.12)	< 0.001
Strongly agree/ Agree	29 (8.1)	16 (55.2)	13 (44.8)	4.40 (1.34–14.48)	0.015
Strongly disagree/ Disagree	315 (88.5)	271 (86.0)	44 (14.0)	1	-
**I have difficulties paying for the medications that are needed to best treat my diabetes**					
Not sure	11 (3.1)	5 (45.5)	6 (54.5)	2.44 (1.36–4.37)	0.003
Strongly agree/ Agree	112 (31.5)	84 (75.0)	28 (25.0)	8.79 (2.52–30.69)	0.001
Strongly disagree/ Disagree	233 (65.4)	205 (88.0)	28 (12.0)	1	-
**I have difficulties getting the supply of medication(s) needed to treat my diabetes**					
Not sure	5 (1.4)	3 (60.0)	2 (40.0)	3.24 (1.57–6.69)	0.001
Strongly agree/ Agree	39 (11.0)	25 (64.1)	14 (35.9)	3.86 (0.63–23.71)	0.145
Strongly disagree/ Disagree	312 (87.6)	266 (85.3)	46 (14.7)	1	-
**I am worried about my financial future due to my diabetes**					
Not sure	17 (4.8)	12 (70.6)	5 (29.4)	3.86 (1.96–7.60)	< 0.001
Strongly agree/ Agree	184 (51.7)	139 (75.5)	45 (24.5)	4.97 (1.50–16.45)	0.009
Strongly disagree/ Disagree	155 (43.5)	143 (92.3)	12 (7.7)	1	-
**My medication(s) to treat diabetes interfere(s) with my normal life**					
Not sure	11 (3.1)	6 (54.5)	5 (45.5)	5.07 (2.77–9.27)	< 0.001
Strongly agree/ Agree	114 (32.0)	77 (67.5)	37 (32.5)	8.79 (2.46–31.38)	0.001
Strongly disagree/ Disagree	231 (64.9)	211 (91.3)	20 (8.7)	1	-
**My family argues with me about how I choose to take care of my diabetes**					
Not sure	8 (2.2)	5 (62.5)	3 (37.5)	2.25 (1.24–4.06)	0.007
Strongly agree/ Agree	87 (24.4)	64 (73.6)	23 (26.4)	3.75 (0.86–16.37)	0.079
Strongly disagree/ Disagree	261 (73.3)	225 (86.2)	36 (13.8)	1	-
**The polyclinic staff has successfully managed my diabetes-related stresses**					
Not sure	44 (12.4)	33 (75.0)	11 (25.0)	0.74 (0.37–1.5)	0.408
Strongly agree/ Agree	246 (69.1)	208 (84.6)	38 (15.4)	1.36 (0.55–3.39)	0.510
Strongly disagree/ Disagree	66 (18.5)	53 (80.3)	13 (19.7)	1	-
**I have ever consulted a psychologist and/or counsellor to work through my diabetes-related stresses**					
Not sure	6 (1.7)	4 (66.7)	2 (33.3)	4.49 (1.45–13.89)	0.009
Strongly agree/ Agree	13 (3.7)	7 (53.8)	6 (46.2)	2.62 (0.47–14.67)	0.273
Strongly disagree/ Disagree	337 (94.7)	283 (84.0)	54 (16.0)	1	-
**Apart from healthcare professionals, there are other persons who I can talk to about my diabetes**					
Not sure	16 (4.5)	11 (68.8)	5 (31.3)	0.94 (0.53–1.68)	0.837
Strongly agree/ Agree	201 (56.5)	168 (83.6)	33 (16.4)	2.18 (0.69–6.84)	0.183
Strongly disagree/ Disagree	139 (39)	115 (82.7)	24 (17.3)	1	-

After adjusting for confounding factors, younger age (AOR (95% CI) = 0.93 (0.89–0.97), *p* = 0.004), ex-smokers (AOR (95% CI) = 22.30 (2.43–204.71), *p* = 0.006), history of kidney disease (AOR (95% CI) = 3.41 (1.39–8.35), *p* = 0.007) and those who felt that medications interfered with their normal lives (AOR (95% CI) = 2.89 (1.38–6.08), *p* = 0.005) remained significantly associated with DRD. A positive PHQ-9 screen (AOR (95% CI) = 4.98 (1.19–20.86), *p* = 0.028) as well as a lower EQ5D index score (AOR (95% CI) = 0.11 (0.01–0.97), *p* = 0.047) continued to be significantly associated with DRD (Table 5).

**Table 5:** Factors affecting diabetes-related distress using logistic regression

	**Adjusted OR (95% CI)**	***p*-value**
**Age**	0.93 (0.89–0.97)	0.001
**Gender**		
Male	0.73 (0.31–1.67)	0.453
Female	1	-
**Smoker**		
Non-smoker	6.48 (0.84–49.79)	0.072
Ex-smoker	22.30 (2.43–204.71)	0.006
Current smoker	1	-
**Experience symptoms of low blood sugar in past 12 months**		
None	1	-
At least once every few months	1.20 (0.55–2.63)	0.651
**Nephropathy and/or chronic kidney disease**		
Yes	3.41 (1.39–8.35)	0.007
No	1	-
**GAD-7**		
< 10	1	-
≥ 10	3.13 (0.91–10.74)	0.069
**PHQ-9**		
< 10	1	-
≥ 10	4.98 (1.19–20.86)	0.028
**EQ VAS**	0.99 (0.97–1.02)	0.572
**EQ5D Index**	0.11 (0.01–0.97)	0.047
**I feel anxious about my weight**		
Strongly agree/ Agree	1.64 (0.79–3.42)	0.188
Not sure/ Strongly disagree/ Disagree	1	-
**I feel discriminated against because I have diabetes**		
Strongly agree/ Agree	2.41 (0.81–7.16)	0.113
Not sure/ Strongly disagree/ Disagree	1	-
**I have difficulties paying for the medications that are needed to best treat my diabetes**		
Strongly agree/ Agree	0.78 (0.32–1.90)	0.585
Not sure/ Strongly disagree/ Disagree	1	-
**I have difficulties getting the supply of medication(s) needed to treat my diabetes**		
Strongly agree/ Agree	2.70 (0.89–8.24)	0.080
Not sure/ Strongly disagree/ Disagree	1	-
**I am worried about my financial future due to my diabetes**		
Strongly agree/ Agree	1.69 (0.73–3.91)	0.218
Not sure/ Strongly disagree/ Disagree	1	-
**My medication(s) to treat diabetes interfere(s) with my normal life**		
Strongly agree/ Agree	2.89 (1.38–6.08)	0.005
Not sure/ Strongly disagree/ disagree	1	-
**My family argues with me about how I choose to take care of my diabetes**		
Strongly agree/ Agree	1.34 (0.61–2.95)	0.462
Not sure/ Strongly disagree/ Disagree	1	-
**I have ever consulted a psychologist and/or counsellor to work through my diabetes-related stresses**		
Strongly agree/ Agree	1.11 (0.25–4.92)	0.894
Not sure/ Strongly disagree/ Disagree	1	-

## Discussion

The DRD prevalence of 17.4% is lower than the 32% previously reported when the PAID questionnaire was administered in a tertiary endocrinology clinic [20]. It is compatible with two other studies where the prevalence in primary care was lower compared to secondary care [7, 8]. PWDs in secondary and tertiary centres tend to be more complex and with more complications compared to those who are treated in the primary care setting. No difference was noted in the number of comorbidities or number of medications between those with and those without DRD in our study. Although the prevalence of DRD seems to be low in primary care setting, the absolute numbers are still significant. Effective interventions to address DRD can potentially result in marked reduction in T2DM-related disease burden.

Subjects with DRD were younger compared to subjects without DRD. This result is consistent with local [32] as well as overseas [6, 9–11] studies. Younger working PWDs face financial stressors, challenges at work and family responsibilities which may increase their difficulties of living with T2DM. They may perceive their illness as a threat or a loss at a time in their lives when they expect themselves to be able-bodied to perform their role as providers or caregivers for their families, hence reacting more negatively to the stressor. Clinicians need to recognise and address the challenges unique to these younger PWDs. A good doctor-patient relationship, based on empathic communication and person-centred approaches, such as motivational interviewing, has been found to reduce diabetes distress, and also improve self-care [17, 33, 34].

Smoking is well known to increase cardiovascular morbidity and mortality among PWDs, and most, if not all, guidelines would recommend that PWDs do not smoke. Our study found that ex-smokers, defined as those who have stopped smoking for at least one year, were at risk for DRD. While most trials take six months to one year as a proxy for life-time smoking cessation, a meta-analysis estimates that annual relapse rates after the first year is approximately 10% [35]. Quitting smoking and remaining smoke-free is challenging, even for those without diabetes. Many who smoke do so as a response to stress. PWDs with DRD who are ex-smokers may benefit from frequent check-ins on their smoking remission status so as to keep them in the maintenance phase of smoking cessation, and to identify and reduce stressors that may trigger a relapse.

The result revealed that PWDs who experienced hypoglycaemia symptoms in the past one year were more likely to have DRD. The perception that diabetes medications interfere with normal life may have been a confounder in this relationship. Hypoglycaemia has been identified as a factor associated with DRD in other multi-centre studies [9, 12]. The DAWN-2 study examined the frequency and severity of symptomatic hypoglycaemia on DRD, and found an association not only with DRD, but also with well-being (WHO-5 Wellbeing Index) and quality of life (WHOQOL-BREF). Hypoglycaemia assessment and management is an integral part of good diabetes care [36]. Managing and educating PWDs about hypoglycaemia, with a focus on addressing issues and concerns related to the PWD’s diabetes medications, not only mitigate this life-threatening risk but may also alleviate their DRD.

DRD, depression and anxiety are often viewed as overlapping concepts, or part of a continuum. However, depression is a separate entity from DRD. Any PWD may have depression or DRD or both. Co et al. reported that DRD was a mediator between poor glycaemic control and health related quality of life [32]. It has also been suggested that the higher rates of depression among PWDs may be mediated by DRD [37]. Thus, psychotherapy and counselling strategies to alleviate DRD in PWD may concurrently prevent and manage their depression and improve their quality of life.

The results identified anxiety around weight and perception that diabetic medications interfered with normal life as significant factors associated with DRD. Younger PWDs could be more image conscious, with higher propensity to be more troubled by weight (and appearance) compared to older PWDs. In a study on self-perception of weight of Korean women aged 20 to 79 years old, it was reported that older women tended to underestimate their weight relative to actual body mass index, compared to younger women [36]. This misperception could reduce incidence of DRD in older PWD but would potentially dampen their activation to lose weight.

The results did not show a significant association between number or type of medications and DRD. While the medications were not free to the PWDs, they were dispensed at highly subsidised prices at polyclinics due to prudent national healthcare finance policies [38], which ensure that local residents will not be deprived of healthcare services due to cost.

DRD is linked to lower health-related QOL [32], and social support has been shown to moderate this relationship [39]. The results of this study support the association between DRD and lower QOL. However, after adjusting for confounders, there was no significant association found between perceived social support and DRD in this study population. In 2016, Singapore’s Ministry of Health declared War on Diabetes, a whole-nation effort to tackle diabetes through various channels, such as public awareness and health promotion campaigns, patient education, and healthcare financing strategies. At the institution level, a multidisciplinary team involving care coordinators, nurses, doctors, pharmacists and medical social workers, provide holistic care through various programmes, including health coaching and telecare services. In the community, organisations such as Diabetes Singapore, provide peer support, counselling and screening for diabetes and diabetes-related complications. While the healthcare structures and social service structures are largely in place, the interface between the two continues to be a focus in the delivery of population health in Singapore.

### Strength and limitations

This is the first study to report the prevalence of DRD in primary care in a cosmopolitan urbanised multi-ethnic Asian community. It will pave the way for the development of appropriate interventions to mitigate DRD among PWDs. A multidisciplinary service has been set up in the institution to address DRD among PWDs, which will be evaluated via health service research methodology.

While this cross-sectional study would not be able to establish causality, the results have identified factors associated with DRD. They include the younger age and comorbidities such as kidney disease. PWDs with such risk factors will be targeted for DRD screening and intervention.

### Implications in clinical practice

In Singapore, the awareness of DRD is slowly gaining pace among primary care physicians. The 2014 Ministry of Health clinical practice guidelines on T2DM recognise DRD as one of the psychosocial issues known to affect self-management and health outcomes of PWDs [40]. Clinical psychologists are now part of the multidisciplinary team in the polyclinics to support the primary care physicians to manage the multifaceted issues faced by PWDs. The effectiveness of such psychologist-led interventions is currently being evaluated.

In the holistic management of PWDs, healthcare professionals are encouraged to recognise the social aspect of living with diabetes, which affects the clinical and psychological outcomes of PWDs. Many of these social factors lie beyond the PWD and the healthcare worker’s control. A first step would be to raise awareness and engage with the larger community that PWDs interact with, to help others understand and empathise with the struggles of PWDs. Public education efforts to raise awareness of DRD and its management should aim to be sustained and far-reaching. The target audience may include immediate family members, such as spouses and children. It can also include managers at the workplace to be more sympathetic and facilitative to the needs of PWDs, for example having suitable meal breaks and hypoglycaemia first aid. Education content can aim to dispel common misconceptions and provide tips to engage friends and family to support the PWDs. Such family-centric and community led services await evaluation to determine their effectiveness in alleviating the disease burden imposed on the PWDs.

The associated factors and patterns of DRD could also change with time as the healthcare system and societal pressures evolve. The tool for DRD assessment has to adapt to the changing healthcare eco-system and needs to be simplified for ease of implementation in routine clinical services.

## Conclusion

One in six (17.4%) PWDs with poor glycaemic control had DRD in this study. Younger age, ex-smoker status, kidney disease, depressive symptoms and the perception that medications interfere with normal life are associated with their DRD. The results highlight the need for primary care physicians to proactively screen PWDs for DRD, especially when their glycaemic control is suboptimal, and to direct them for evidence-based therapy. Beyond glycaemic control, the mental and emotional health of PWDs (and their families) must not be overlooked, as it will impact their health outcomes and quality of life.

## Supplementary Information

**Additional file 1: Annex 1.** Problem Area In Diabetes questionnaire.

## Data Availability

The datasets used and analysed in this study are not publicly available but are available from the corresponding author upon reasonable request.
