# Supplementary material for: Factors associated with diabetes-related distress among Asian patients with poorly controlled type-2 diabetes mellitus: a cross-sectional study in primary care
Source: BMC Prim Care. 2023 Feb 27;24:54. doi: 10.1186/s12875-023-02012-w (PMC9969642; doi:10.1186/s12875-023-02012-w)
Supplement: Supplementary file 1 — Additional file 1: Annex 1. Problem Area In Diabetes questionnaire. [file 12875_2023_2012_MOESM1_ESM.docx]

Annex 1: Problem Area In Diabetes questionnaire

**Which of the following diabetes issues are currently a problem for you?**

*(Circle the number that gives the best answer for you. Please provide an answer for each question.)*

|  | *0= Not a problem*  *1= Minor problem*  *2= Moderate problem*  *3= Somewhat serious problem*  *4= Serious problem* | Not  a problem | Minor problem | Moderate problem | Somewhat serious problem | Serious problem |
| --- | --- | --- | --- | --- | --- | --- |
| 1. | Not having clear and concrete goals for your diabetes care? | 0 | 1 | 2 | 3 | 4 |
| 2. | Feeling discouraged with your diabetes treatment plan? | 0 | 1 | 2 | 3 | 4 |
| 3. | Feeling scared when you think about living with diabetes? | 0 | 1 | 2 | 3 | 4 |
| 4. | Uncomfortable social situations related to your diabetes care (e.g. people telling you what to eat)? | 0 | 1 | 2 | 3 | 4 |
| 5. | Feelings of deprivation regarding food and meals? | 0 | 1 | 2 | 3 | 4 |
| 6. | Feeling depressed when you think about living with diabetes? | 0 | 1 | 2 | 3 | 4 |
| 7. | Not knowing if your mood or feelings are related to your diabetes? | 0 | 1 | 2 | 3 | 4 |
| 8. | Feeling overwhelmed by your diabetes? | 0 | 1 | 2 | 3 | 4 |
| 9. | Worrying about low blood sugar reactions? | 0 | 1 | 2 | 3 | 4 |
| 10. | Feeling angry when you think about living with diabetes? | 0 | 1 | 2 | 3 | 4 |
| 11. | Feeling constantly concerned about food and eating? | 0 | 1 | 2 | 3 | 4 |
| 12. | Worrying about the future and the possibility of serious complications? | 0 | 1 | 2 | 3 | 4 |
| 13. | Feelings of guilt or anxiety when you get off track with your diabetes management? | 0 | 1 | 2 | 3 | 4 |
| 14. | Not “accepting” your diabetes? | 0 | 1 | 2 | 3 | 4 |
| 15. | Feeling unsatisfied with your diabetes physician? | 0 | 1 | 2 | 3 | 4 |
| 16. | Feeling that diabetes is taking up too much of your mental and physical energy every day? | 0 | 1 | 2 | 3 | 4 |
| 17. | Feeling alone with your diabetes? | 0 | 1 | 2 | 3 | 4 |
| 18. | Feeling that your friends and family are not supportive of your diabetes management efforts? | 0 | 1 | 2 | 3 | 4 |
| 19. | Coping with complications of diabetes? | 0 | 1 | 2 | 3 | 4 |
| 20. | Feeling “burned out” by the constant effort needed to manage diabetes? | 0 | 1 | 2 | 3 | 4 |

Copyright (c) 1999-2016 by Joslin Diabetes Center.  All rights reserved.  Reprinted with permission.
